# Understanding the Function Constitution and Influence Factors on Communication for the WeChat Official Account of Top Tertiary Hospitals in China: Cross-Sectional Study

**DOI:** 10.2196/13025

**Published:** 2019-12-09

**Authors:** Lining Shen, Shimin Wang, Wenqiang Chen, Qiang Fu, Richard Evans, Fuqiang Lan, Wei Li, Juan Xu, Zhiguo Zhang

**Affiliations:** 1 School of Medicine and Health Management Tongji Medical College Huazhong University of Science & Technology Wuhan China; 2 Hubei Provincial Research Center for Health Technology Assessment Wuhan China; 3 Institute of Smart Health Huazhong University of Science & Technology Wuhan China; 4 Department of Epidemiology and Biostatistics, College for Public Health and Social Justice Saint Louis University St Louis, MO United States; 5 College of Engineering, Design and Physical Sciences Brunel University London London United Kingdom

**Keywords:** WeChat official account, WeChat service account, social media, function constitution, tertiary hospital, tertiary care centers, health care, WeChat communication index, mobile health, telemedicine

## Abstract

**Background:**

Widespread adoption and continued developments in mobile health care technologies have led to the improved accessibility and quality of medical services. In China, WeChat, an instant messaging and social networking app released by the company Tencent, has developed a specific type of user account called WeChat official account (WOA), which is now widely adopted by hospitals in China. It enables health care providers to connect with local citizens, allowing them to, among other actions, send regular updates through mass circulation. However, with the diversity in function provided by WOA, little is known about its major constitution as well as the influence factors on the WeChat communication index (WCI). The WCI has been widely used in social media impact ranking with various types of WeChat content to fully reflect the dissemination and coverage of tweets as well as the maturity and impact of WOA.

**Objective:**

There are two typical WOAs available to users, namely, WeChat subscription account (WSSA) and WeChat service account (WSVA). The biggest difference between them is the frequency of messages transmitted. This study aimed to explore the function constitution of WSVA adopted by top tertiary hospitals in China and the major contributors of the WCI score.

**Methods:**

A total of 681 top tertiary hospitals were selected from the Hospital Quality Monitoring System; the WOA of every top tertiary hospital was retrieved in the WeChat app. We divided core functional items of WSVAs using categorical principal component analysis. To elicit the factors that influenced the use of WSVA, quantile regression was employed to analyze the WCI score.

**Results:**

From the 668 WOAs identified, adoption of WSVAs (543/668, 81.3%) was more than that of WSSAs (125/668, 18.7%). Functional items of WSVAs were categorized into four clusters: (1) hospital introduction, (2) medical services, (3) visiting assistants, and (4) others. With regard to the influence factors on the WCI, the impact of the activity index of WSVA and the total visiting number of outpatients and emergencies on WCI were statistically significant and positive in all quantiles. However, the year of certification, the type of hospital, the year of public hospital reform, and the number of beds merely affected the WCI at some quantiles.

**Conclusions:**

Our findings are considered helpful to tertiary hospitals in developing in-depth functional items that improve patient experience. The tertiary hospitals should take full advantage of times of posting and provide high-quality tweets to meet the various needs of patients.

## Introduction

### Background

Mobile technologies and internet-connected devices hold great potential for improving health care delivery and services [1,2]. According to a report released by the Business Communications Company in 2017, the global market for mobile health technologies is expected to grow at an annual rate of 28.6% [3]. Among others, social media, an important part of mobile apps, is defined by Kaplan and Haenlein [4] as “a set of Internet-based applications built on Web 2.0 ideas and technologies that allow for the creation and exchange of user-generated content.” Social media has changed the nature of interaction between individuals and health care providers. It also provides a medium for the public, patients, and health care professionals to communicate on health care issues [5].

With developments in internet capabilities, such as speed and widespread connectivity, social media has continued to manifest its influence. An increasing amount of research involved in the effects of social media on the health care industry has been observed [6-8]. Some researchers have evaluated the academic influence of WeChat [9]. Gan [10] conducted a survey to explore the adoption of WeChat in Chinese public libraries. In the health care field, many researchers have investigated the application of social media within and outside hospitals (eg, hospital apps [11,12], health care apps [13], Facebook [14,15], and WeChat [16]). Others have reported interventions and assistance using social media for particular patients (eg, orthodontic patients [17] and glaucoma patients [18]). In addition, some scholars have concentrated on the use of health care apps by older Germans [19].

### WeChat Official Account and WeChat Communication Index

WeChat is the most popular social media mobile app in China, providing over 1 billion daily active users with a multipurpose messaging and mobile payment app [20,21]. The app has rapidly become an integral part of people’s daily life, with citizens now using it to message, share updates, find people in their local community, transfer money, and pay utility bills in real time [22]. One of the main accounts available to users on WeChat, since 2012, is the *WeChat official account (WOA)*, which is a lightweight app on WeChat and which targets celebrities, government agencies, and businesses to facilitate cooperation and promotion. WOA transmits messages, including real-time adverts to WeChat users, thus reducing propagation costs and raising brand popularity, among others. According to a report issued by iResearch in 2015, 79.2% of WeChat users subscribe to certain WOAs [23].

There are 2 typical kinds of WOAs: WeChat subscription account (WSSA) and WeChat service account (WSVA). WSSA provides a means to reach the subscribers by information propagation. In contrast, WSVA provides entities, such as governments and businesses, with more powerful business services and user management capabilities. One of the main differences between the 2 accounts is frequency of message transmission. Specifically, WSSA can send out 1 group of messages every day, whereas WSVA can only send out 4 groups of messages every month [24].

The WeChat communication index (WCI), released in March 2017 as an integral feature of the WeChat app, provides users with the ability to measure the popularity of keywords based on their search volume and appearance within articles or shared stories [25], which is currently one of the most important media reports in China. Users can track the popularity of keywords over a 7-, 30-, or 90-day period, identifying a WCI score. The WCI score is derived from a series of complex and rigorous calculation formulas, which consist of 4 dimensions, that is, *overall communication power,* *average dissemination power,* *headline communication power,* and *peak dissemination power* [26] (for the detailed calculation formula of WCI, see Multimedia Appendix 1). Owing to its comprehensiveness, transparency, and openness, the WCI has high credibility and authority in the Chinese industry and has been widely used in various types of social media impact ranking of WeChat content. The WCI can fully reflect the dissemination and coverage of messages released by WOAs as well as the maturity and impact of WOAs, with it currently being adopted by over 20,000 institutions. Besides, WCI has been updated over time, with the latest version being 13.0 at present.

### Classification and Reform for Public Hospital in China

According to the *Regulation of Medical Institutions*, released by the National Health Commission of the People’s Republic of China (NHC-PRC) [27], there are different types of medical institutions, including comprehensive hospitals (CHs), specialized hospitals (SHs), maternal and child health center, integrative traditional Chinese and western medicine hospitals, township health centers, and ethnicity hospitals. The CHs and SHs play a leading role in China in terms of the number and scale of medical services provided; the main difference between them is that the CHs have complete departments, whereas the SHs only provide one or a few medical specialties. Furthermore, hospitals in China are classified into 3 tiers according to the present Hospital Grading System: tier 1 (primary), tier 2 (secondary), and tier 3 (tertiary), each of which is further divided into 3 levels, including level 1, level 2, and level 3 [28]. The tertiary hospitals with level 3 represent the most advanced level of service provided in China. In addition, the diction *tertiary hospitals with level 3* is simplified as *top tertiary hospitals* in some literature [29-31].

According to *Guiding Opinions on the Pilot Reform of Public Hospitals*, released by NHC-PRC [32], the hospital reform was phased in and implemented in all public hospitals in 5 consecutive batches from 2010. The reform endeavored to improve the availability, accessibility, quality, and efficiency of medical services in public hospitals. The application of WSVA and the derived WCI was related to and contributed to fulfilling these purposes.

### Objectives

WOAs are now seen as an essential marketing and branding tool by government agencies, celebrities, and businesses alike. Citizens in China are connecting with their favorite brands to receive real-time updates and important information, making it a crucial element of everyday life, with no exception in the health care field in China [33,34]. It is perceived as an ideal medium for implementing a *patient-centered* approach to health care. Specifically, the WOA of electrocardiogram remote consultation for township health center was established in Guandong province in China, through which timely medical service can be provided to address poor diagnostic quality in primary health institution [35]. The design and establishment of a mobile medical consumables management system based on WOA is helpful to manage medical consumable materials more conveniently and efficiently [36]. However, much remains unknown with regard to the main functional items and impact factors on the WCI for WOAs. To improve our understanding of the use of WOAs by top tertiary hospitals in China, the following 3 research questions are identified:

1. What was the status quo regarding the application of WOAs used by the top tertiary hospitals in China?
2. What was the major function constitution of WOA?
3. What were the factors that influence the WCI and their effects on WCI at different levels?

## Methods

### Sample and Data Collection

**Figure 1:**
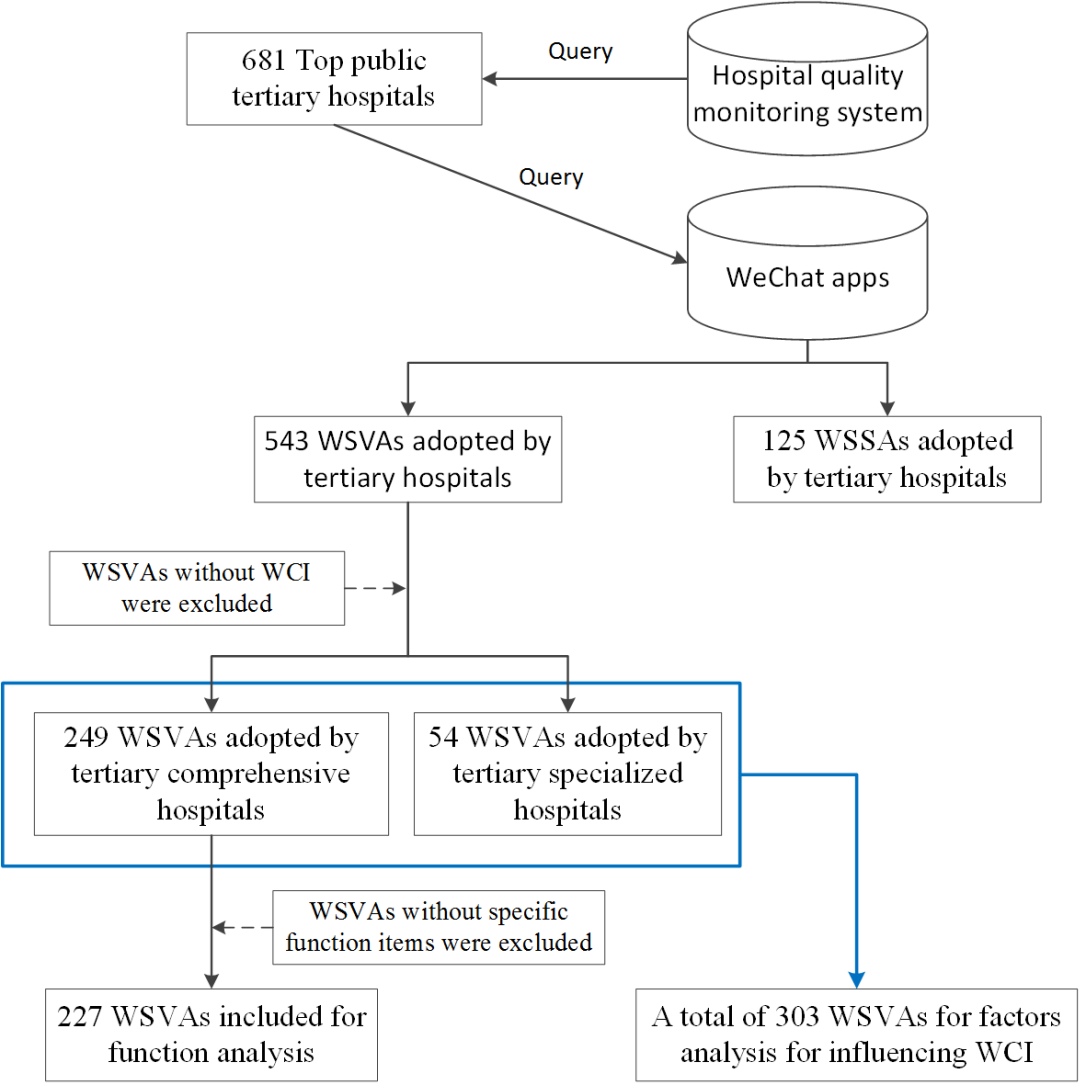
Process for identifying the WeChat official accounts adopted by tertiary hospitals. WCI: WeChat communication index; WSSA: WeChat subscription account; WSVA: WeChat service account.

The process used for identifying the WOAs of top tertiary hospitals in China is shown in Figure 1. Specifically, 3 stages were completed, as follows, regarding the data collection of this study.

First, through interrogation of the Hospital Quality Monitoring System developed by NHC-PRC [37], 681 top tertiary, comprehensive or specialized, hospitals were identified on May 3, 2018. The hospitals were then classified into different groups based on different regions of China [38], that is, Western China (WC), Central China (CC), and Eastern China (EC), which corresponds to a gradually incremental population density and economic development level. Among the top tertiary hospitals, each of 3 research assistants had access to 227 (681 divided by 3) official websites. They collected the *number of beds* and *total visiting number of outpatients and emergency room* in 2017 from each hospital official website and confirmed whether each website of tertiary hospital presented the official quick response (QR) code of WOA. The number of beds mainly refers to the number of open beds representing the capacity of the hospital, which is counted in thousands. However, the number of authorized beds, which relates to the governmental subsidy to each public hospital, was used to replace the number of open beds when the latter could not be found. Moreover, the total visiting number of outpatients and emergencies reflects the quality of medical service to some extent. The research assistants used smartphones to search and subscribe to the WOAs of each hospital to ascertain the type and the year of certification. As a result, 543 WSVAs and 125 WSSAs were identified and verified for further analysis.

Second, 3 research assistants searched these WSVAs on the official website of the Qingbo Index, obtaining the average WCI, number of posting times, and the total number of posted tweets, for each WSVA in April 2018. Here, the average WCI was the sum of the WCI generated by each posting divided by the number of posting times in April 2018. Considering that most WSSAs have no WCI, 303 WSVAs, including 249 WSVAs adopted by tertiary CHs, were chosen for subsequent analysis.

Finally, as for 249 WAVAs, these 3 research assistants randomly chose a certain province located in each region in China and subscribed to all WSVAs in the province, and thereafter, they listed all the specific functional items of each WSVA. As some WSVAs have no specific functional items, a total of 227 WSVAs were identified from 249 WSVAs for function analysis. Then, a list of 15 functions of WSVAs was identified, following discussions and resolution of any discrepancies or disagreements. Eventually, 1 research assistant proceeded to record the functional items of the remaining WSVAs. In addition, 303 WSVAs were employed for analysis of influence factors on the WCI.

### Measure of Factors Influencing the WeChat Communication Index

We investigated 6 indicators: (1) the year of certification of the WSVA, (2) the type of hospital, (3) the year of public hospital reform involved, (4) the number of beds, (5) the total number of visiting outpatients and emergency patients, and (6) the activity index of the WSVA. The impact of these indicators on the WCI was considered. Herein, the certification of the WSVA is optional and officially authorized and approved by the Tencent company. In addition, the certification is not free. After certification is granted, more rights of the WSVA will be given to the owner, which is helpful for the development and utilization of the functional items of the WSVA. We used the following formula to calculate the activity index of each WSVA [9], which partly represents the emphasis from the tertiary hospitals on WOA and is conducive to promoting users’ stickiness:

Activity index = 0.5*ln (number of posting times a month + 1) + 0.5*ln (number of posted tweets a month + 1)

In addition, the variance inflation factor of each independent variable is less than 2. All independent variables and their descriptions and references are listed in Table 1.

**Table 1:** Description of variables that influence WeChat communication indexes.

Variable name	Description and coding	References
CertificationYear	The initial year of certification of the WSVA^a^. The coding is listed as follows: 1=never certified, 2=certification during 2014 to 2016, 3=certification in 2017, and 4=certification in 2018	Gan, 2016 [10], Xu et al, 2015 [39], and Zhang, 2015 [40]
HospitalType	The type of hospital. The coding is listed as follows: 1=specialized hospital and 2=comprehensive hospital	Huang et al, 2019 [41], Chen et al, 2009 [42], Tatro et al, 2019 [43], and Plantier et al, 2017 [44]
ReformYear	The year of public hospital reform involvement. The coding is listed as follows: 1=2010, 2=2014, 3=2015, 4=2016, and 5=2017	Lin et al, 2014 [45]
BedNumber	The number of beds per top tertiary hospital	Lin et al, 2014 [45]
TotalVistingNumber	The total number of visiting outpatients and emergency patients in 2017	Fuller et al, 2019 [46]
ActivityIndex	The activity index of the WSVA	Zhao et al, 2017 [9]

^a^WSVA: WeChat service account.

### Method of Visualization for Distribution of WeChat Official Accounts of Tertiary Hospitals

EChart is an open-source visualization library, implemented in JavaScript, that provides intuitive, highly customizable data visualization charts [47]. We developed a program based on EChart library, using the Java language, which demonstrated the geographical distribution of the WOAs of tertiary hospitals in China. As the map produced is 3 dimensional, the number of WOAs in each province in China is displayed together with the color depth and height of the pillars. Furthermore, the yellow, green, and blue portions of the map represent WC, CC, and EC, respectively.

### Statistical Analysis

#### Function Coding and Categorical Principal Component Analysis

As for each functional item, we coded *1* for having such functional item in WSVA and *2* for having no such function. Then, categorical principal components analysis (CATPCA), a variable-centered method aimed to correlate variables based on the relationship between each other, was used to reduce the dimensionality while accounting for as many of the patterns of variation as possible [48,49]. In CATPCA, categorical variables are optimally quantified in the specified dimensionality. As a result, nonlinear rather than linear relationships between variables can be modeled. The CATPCA was completed using SPSS 20.0 (IBM). In addition, the function constitution presents what functions 227 WSVAs of top tertiary CHs provide and what categories these functions can be roughly divided into using CATPCA.

#### Quantile Regression

Quantile regression, as introduced by Koenker and Bassett, is used to extend the explanation of the conditional mean in ordinary least squares (OLS) regression to the estimation of conditional quantile functions models in which conditional quantiles of the response variable are expressed as functions of observed covariates [50-52]. One advantage of quantile regression, relative to the OLS regression, is that the quantile regression estimates are more robust against outliers in the response measurements, so as to allow a nonnormally distributed response variable. As such, quantile regression could identify heterogeneous effects of independent variables on different conditional quantiles. Therefore, quantile regressions are gradually being recognized as a helpful technique in the case of skewed (non-normal) distribution, which can explain response measurement more accurately and comprehensively than classical methods [53]. The conditional quantile function of *y_i_* given *x_i_* can be specified as *Q_τ_*(*y_i_* | *x_i_*) = *x_i_β_τ_*, with *Q_τ_*(*y_i_ | x_i_*) being the conditional quantile function at quantile *τ*, with 0 <*τ*<1, and *β_τ_* representing the vector of parameters to be estimated [54].

Given that the mean WCI score of 303 WSVAs was 510.67 (SD 220.61) and that WCI did not satisfy normal distribution, with the *P* value of Shapiro-Wilk normality test being .03, we analyzed the factors influencing the WCI using quantile regression performed by R 3.5.1, which offers several packages implementing quantile regression analysis. We now have 11 covariates, plus an intercept. For each of the 12 coefficients, we plotted the 9 distinct quantile regression estimates for τ=(0.1, 0.2, 0.3, 0.4, 0.5, 0.6, 0.7, 0.8, and 0.9) as the black solid curve with filled dots as well as the relevant 95% interval estimates shown by the shaded gray area. For each covariate, the point estimates may be interpreted as the impact of a 1-unit change of the covariate on the relevant conditional WCI quantile, holding other covariates fixed. Thus, each of the plots has a horizontal quantile of the WCI, or τ scale, and the vertical scale indicates the covariate effect. Meanwhile, the red solid line and dashed line in the visualization map of quantile regression analysis show the OLS point, and 95% CI estimates on the conditional mean were reported. Where the shadow area intersects with the black solid line, it suggests that the effect of the covariate on the relevant conditional WCI is not statistically significant because the coefficient corresponding to that quantile is no different from zero.

## Results

### Basic Statistical Information

#### Distribution for Top Tertiary Hospitals

Among the 681 tertiary hospitals, there are 145 SHs and 536 CHs. As shown in Table 2, 307 hospitals were located in EC, accounting for nearly half of the total. Obviously, there exist geographical differences in the distribution of tertiary hospitals.

**Table 2:** Distribution of top tertiary hospitals in different types and regions.

Type of region	Specialized hospital (N=145), n (%)	Comprehensive hospital (N=536), n (%)	Total (N=681), n (%)
Western China	31 (21.4)	130 (24.3)	161 (23.6)
Central China	38 (26.2)	175 (32.6)	213 (31.3)
Eastern China	76 (52.4)	231 (43.1)	307 (45.1)

#### Distribution of WeChat Official Accounts Adopted by Top Tertiary Hospitals

A total of 668 hospitals hold WOAs, accounting for 98.0% (668/681) of the total number of top tertiary hospitals (for the list of WOAs in all provinces of China, see Multimedia Appendix 2). This shows that most top tertiary hospitals attach great importance to the role of their WOAs. In addition, we found that there are merely 397 hospitals that provide QR codes of their WOAs on the official hospital website, showing that some top tertiary hospitals are still lacking the publicity of their WOAs.

**Figure 2:**
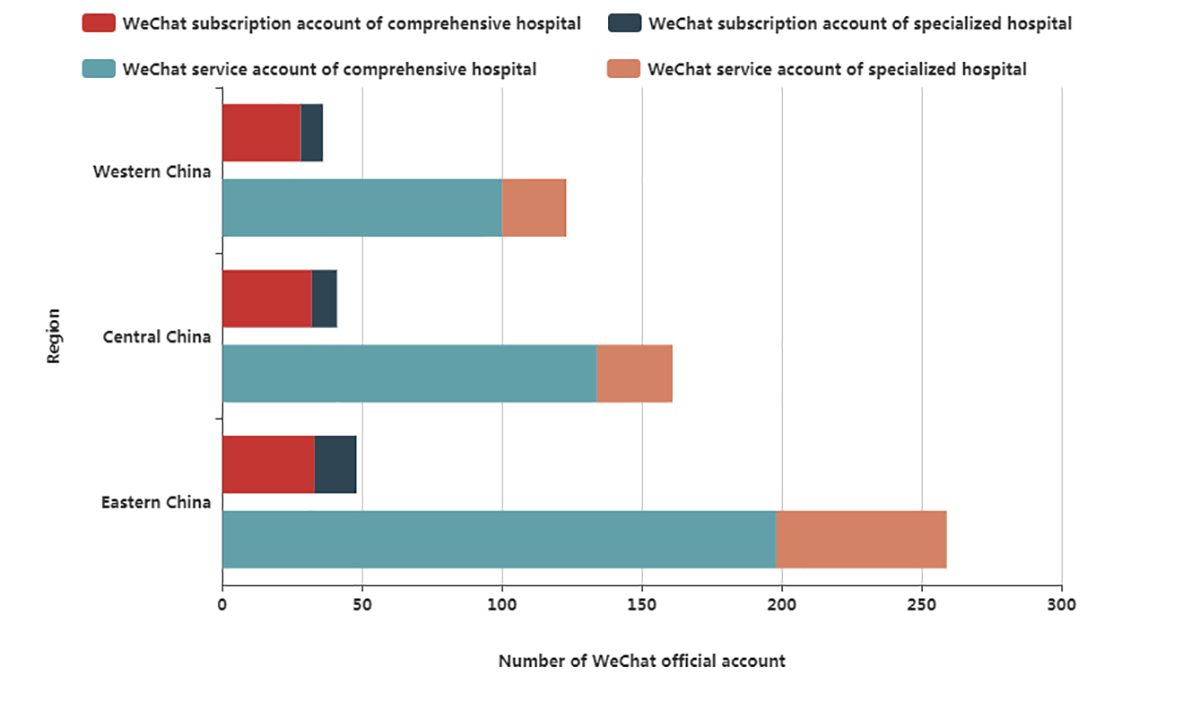
Distribution of WeChat official accounts operated by top tertiary hospitals.

**Figure 3:**
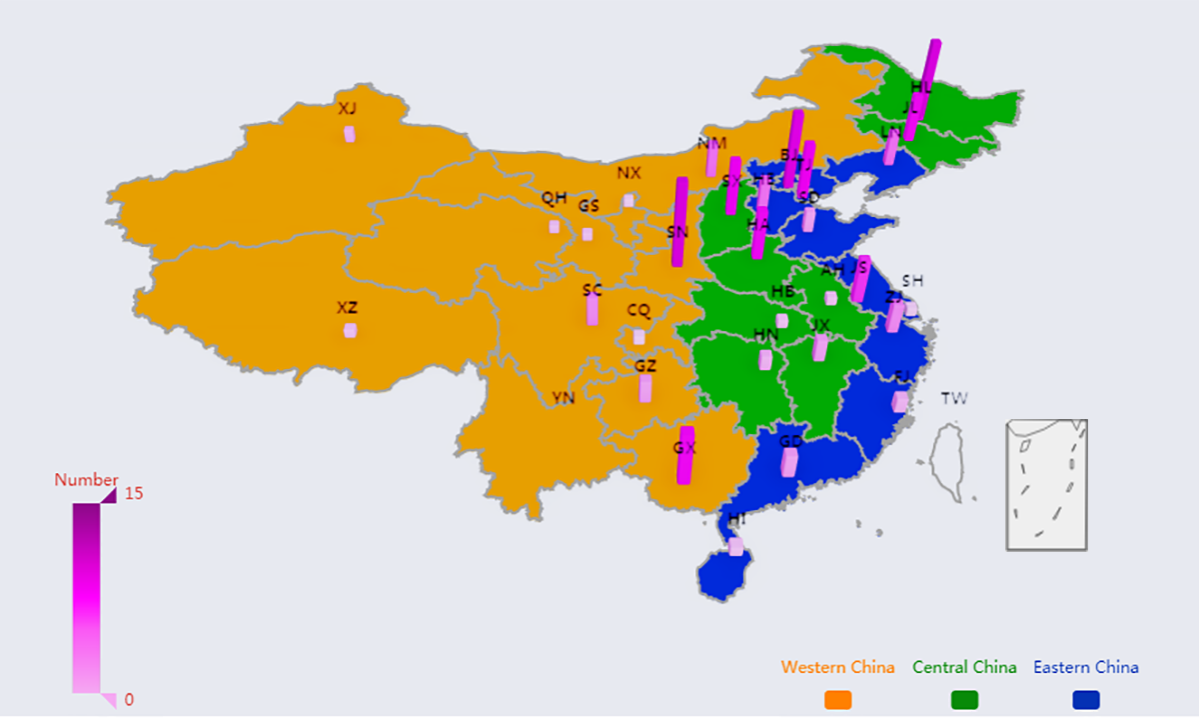
Spatial distribution of WeChat subscription accounts operated by top tertiary hospitals.

**Figure 4:**
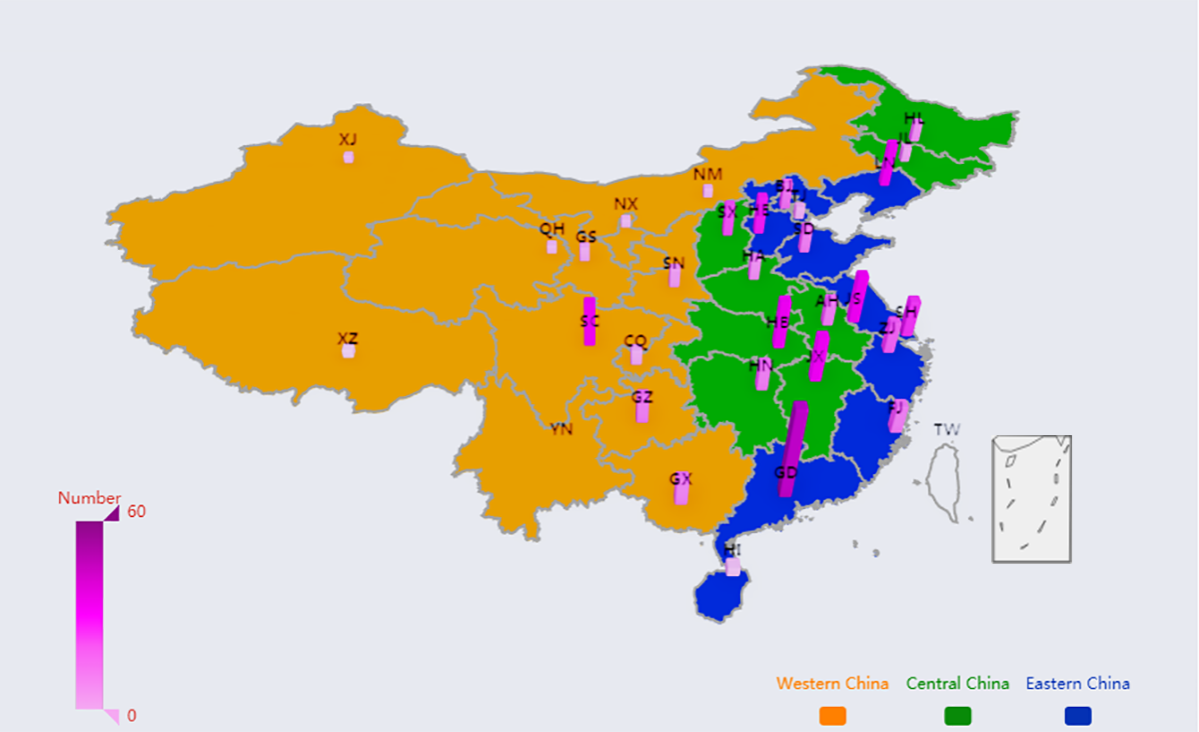
Spatial distribution of WeChat service accounts operated by top tertiary hospitals.

As shown in Figure 2, the EC held 307 WOAs, accounting for nearly half (307/668, 46.0%) of the total of tertiary hospitals, followed by the CC and the WC. Meanwhile, 259 WSVAs adopted by top tertiary hospitals were located in the EC, accounting for 47.7% (259/543) of the total of tertiary hospitals, followed by the CC and the WC. However, the number of WSSAs in the 3 regions is roughly the same. In total, the number of WSSAs or WSVAs adopted by CHs is more than that of SHs because there are more CHs in our dataset. Figures 3 and 4 display the spatial distribution of the sample throughout mainland China. The number of WSVAs is mainly concentrated in the CC and EC. Guangdong province holds the largest number of WSVAs.

### Function Analysis for WeChat Service Account Adopted by Top Tertiary Comprehensive Hospitals in China

#### Function Distribution for WeChat Service Account

Table 3 summarizes the distribution of 15 functional items available in the 227 WSVAs of top tertiary hospitals in China. The average function number of each tertiary hospital is 7.44. It can be seen that the most widely available function across the different regions of China is visiting appointment, followed by the function inquiry, suggesting that most top tertiary hospitals give priority to appointments and patient inquiries. However, the function online consultation was still underdeveloped (17/227, 7.5%) in most top tertiary hospitals.

**Table 3:** Function distribution of WeChat service account operated by top tertiary hospitals.

Function item	Western China (N=41), n (%)	Central China (N=71), n (%)	Eastern China (N=115), n (%)	Total (N=227), n (%)
Hospital brief	24 (59)	50 (70)	65 (56.5)	139 (61.2)
Introduction of department and experts	15 (37)	34 (48)	56 (48.7)	105 (46.3)
Information bulletin	21 (51)	29 (41)	59 (51.3)	109 (48.0)
Medical guide	18 (44)	35 (49)	65 (56.5)	118 (52.0)
Hospital navigation	16 (39)	22 (31)	47 (40.9)	85 (37.4)
Visiting appointment	36 (88)	65 (92)	98 (85.2)	199 (87.7)
Inquiry	28 (68)	53 (75)	92 (80.0)	173 (76.2)
Medical charge payment	18 (44)	36 (51)	68 (59.1)	122 (53.7)
Online consultation	3 (7)	7 (10)	7 (6.1)	17 (7.5)
Intelligent guidance	13 (32)	24 (34)	23 (20.0)	60 (26.4)
Personal information management	27 (66)	51 (72)	87 (75.7)	165 (72.7)
Health education	14 (34)	28 (39)	36 (31.3)	78 (34.4)
Advice and feedback	17 (41)	31 (44)	52 (45.2)	100 (44.1)
Related links	17 (41)	35 (49)	67 (58.3)	119 (52.4)
Others	14 (34)	28 (39)	59 (51.3)	101 (44.5)

#### Function Categorization for WeChat Service Accounts

Owing to the insufficient employment of the function *online consultation*, we used the remaining 14 functional items for the subsequent analysis using CATPCA. Notice that the eigenvalue for a dimension should be larger than 1, when all variables are single nominal. Therefore, there was a 4-dimensional solution, accounting for 50.06% of the total variance (for more details, see Multimedia Appendix 3).

**Figure 5:**
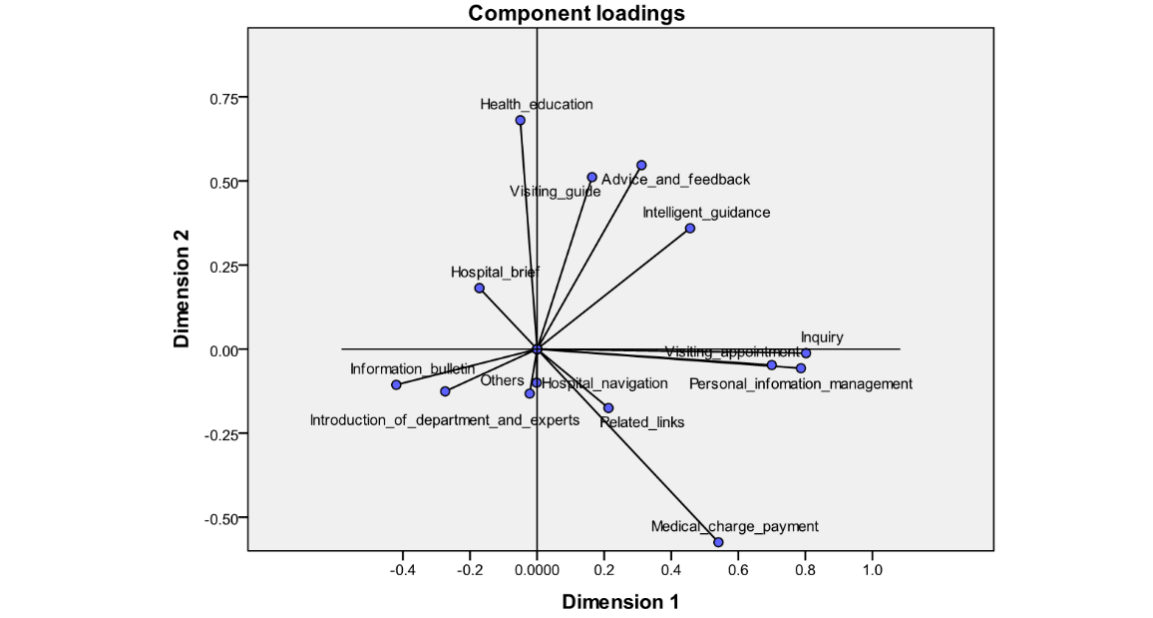
Component loadings of 14 functional items of WeChat service accounts between dimensions 1 and 2.

**Figure 6:**
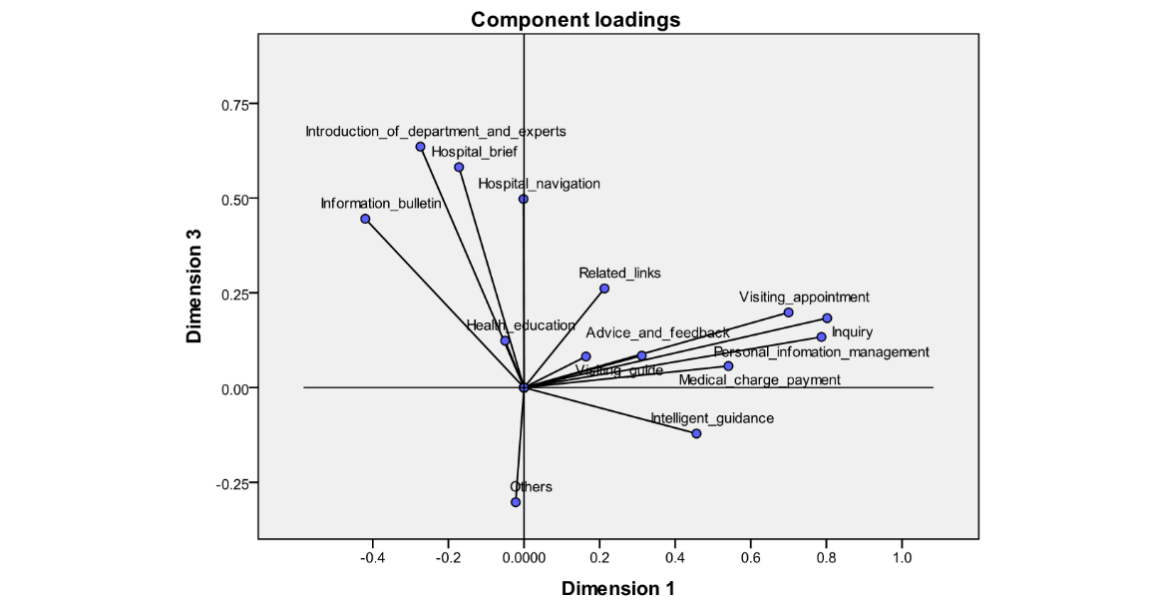
Component loadings of 14 functional items of WeChat service accounts between dimensions 1 and 3.

To form a clearer observation, the plane coordinate maps were chosen and drawn for dimensions 1 and 2, and dimensions 1 and 3, respectively, as shown in Figures 5 and 6. As can be seen, 14 functional items could be categorized into 4 clusters by using CATPCA, each of which refers to the adoption of functional items of the WSVA at the same time, owing to the higher correlation within each cluster. The name of each cluster was refined based on the functional items in the respective cluster.

As shown in Figure 5, cluster 1 is located in quadrant I with the name of visiting assistance, which contains visiting guide, advice and feedback, and intelligent guidance. Cluster 2 is referred to as medical services, covering inquiry, personal information management, visiting appointment, medical charge payment, and related links, all of which are present in quadrant IV. Cluster 3, named hospital introduction, is in quadrant II, which includes the hospital brief, introduction of department and expert, information bulletin, hospital navigation, and health education. Cluster 4 is in quadrant III, which merely holds 1 functional item *others*, as shown in Figure 6.

### Influence Factors on the WeChat Communication Index

#### Descriptive Statistics for Influence Factors

The tertiary hospitals with the 10 highest WCIs are shown in Table 4. As seen, 6 of these WSVAs of tertiary hospitals were certificated in 2018. There are 8 CHs and 2 SHs. In terms of regional distribution, 9 tertiary hospitals are located in EC and CC, except 1, which is based in WC. Except from the 2 hospitals with low activity index, which are affiliated to Sun Yat-sen University, the remaining 8 hospitals have a high activity index. The *West China Hospital, Sichuan University* ranks highest, far ahead of other tertiary hospitals.

The basic information for independent variables is presented in Table 5. It is observed that most WSVAs operated by top tertiary hospitals were certificated in 2017 or 2018, making up 94.4% (287/303) of the total. CHs comprised 79.5% (241/303) of the total. Most hospitals (163/303, 53.8%) are located in EC. As of 2017, all tertiary hospitals were involved in public hospital reform.

**Table 4:** Top 10 WeChat communication indexes of top tertiary hospitals.

No	Name of top tertiary hospital	Average WCI^a^	Certification year	Hospital type	Region type	Activity index
1	West China Hospital, Sichuan University	1233.49	2014	CH^b^	WC^c^	2.004
2	Hunan Children’s Hospital	1099.34	2018	SH^d^	CC^e^	2.087
3	The First Affiliated Hospital, Sun Yat-sen University	1074.46	2018	CH	EC^f^	1.498
4	Guangzhou Women and Children’s Medical Center	1026.92	2017	SH	EC	1.966
5	Tongji Hospital, Tongji Medical College, Huazhong University of Science & Technology	1012.37	2017	CH	CC	2.047
6	Qilu Hospital of Shandong University	996.97	2018	CH	EC	2.124
7	The Third Affiliated Hospital, Sun Yat-sen University	991.86	2017	CH	EC	0.693
8	The First People’s Hospital of Foshan	981.47	2018	CH	EC	2.465
9	Yichang Central People’s Hospital	951.52	2018	CH	CC	2.538
10	Shanghai Ninth People’s Hospital, School of Medicine, Shanghai JiaoTong University	949.48	2018	CH	EC	2.159

^a^WCI: WeChat communication index.

^b^CH: comprehensive hospital.

^c^WC: Western China.

^d^SH: specialized hospital.

^e^CC: Central China.

^f^EC: Eastern China.

**Table 5:** Basic information for independent variables influencing WeChat communication index.

Variable name		Values
**CertificationYear (N=303), n (%)**		
	Never certificated (reference category)	7 (2.3)
	Certification during 2014 to 2016 (CertificationYear1)	9 (3.0)
	Certification in 2017 (CertificationYear2)	173 (57.1)
	Certification in 2018 (CertificationYear3)	114 (37.6)
**HospitalType (N=303), n (%)**		
	Specialized hospital (reference category)	62 (20.5)
	Comprehensive hospital (HospitalType1)	241(79.5)
**ReformYear (N=303), n (%)**		
	Involved in 2010 (reference category)	51 (16.8)
	Involved in 2014 (ReformYear1)	30 (9.9)
	Involved in 2015 (ReformYear2)	74 (24.4)
	Involved in 2016 (ReformYear3)	54 (17.8)
	Involved since 2017 (ReformYear4)	94 (31.0)
BedNumber, mean (SD)		1.83 (1.06)
TotalVisitingNumber, mean (SD)		153.09 (116.43)
ActivityIndex, mean (SD)		1.99 (0.45)

#### Quantile Regression Analysis for WeChat Communication Index

**Figure 7:**
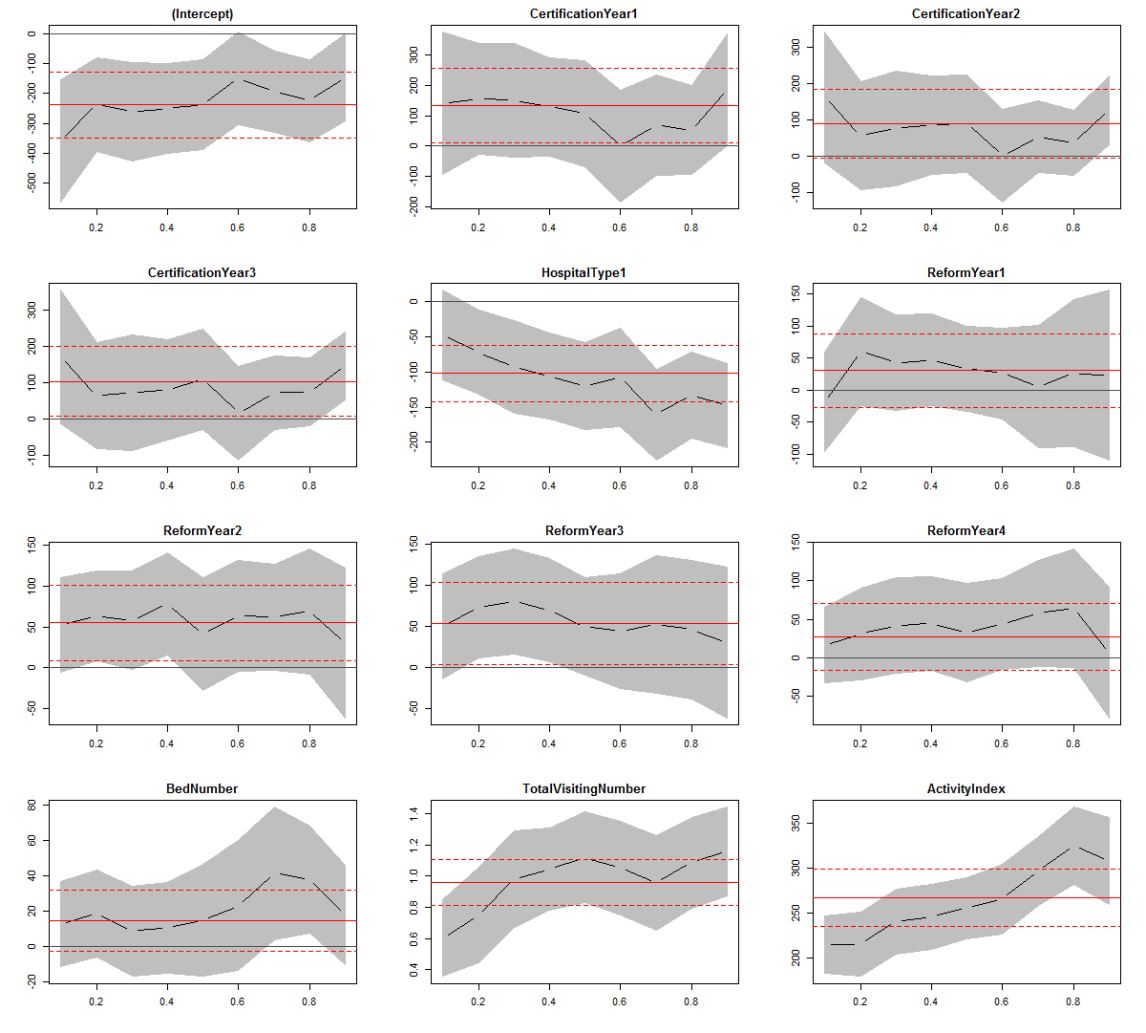
Ordinary least squares and quantile regression estimates for WeChat communication index model. Vertical axes show coefficient estimates of named explanatory variable; horizontal axes depict the quantiles of the WeChat communication index variable; the red plain line and the red dashed line represent the ordinary least squares point and 95% CI estimates on the conditional mean, respectively; the black dashed lines represent conditional quantile estimates.

A summary of the quantile regression results for WCI, with each quantile in 0.10 increments, is shown in Figure 7 (for more detailed results of quantile regression, see Multimedia Appendix 4). The dummy variables, including the year of certification of WSVA, the type of tertiary hospital, and the year of public hospital reform involved, were generated. In general, it can be seen that all dummy variables did not have a statistically significant effect on WCI, except the type of tertiary hospital, although there were some significant effects on WCI at some quantiles.

Specifically, we took uncertification as the reference category of the year of certification for WSVA. The impact of the different years of certification on WCI was seen as positive, but insignificant, with a relatively less fluctuation across the different quantiles of WCI. CHs are seen to have a negative impact on WCI, in comparison with SHs, with a downward trend as the quantile increases. In essence, the higher the WCI was, the bigger the effect difference on WCI between CH and SH had. Namely, SHs place more emphasis on WSVA than CHs. It should also be noted that the type of tertiary hospital has a significant impact on WCI after 0.20 quantile and beyond. The impact of the year of the public hospital reform involved on WCI takes 2010 as the reference category; the impact on WCI is generally positive in different periods because one of the purposes of the reform in public hospitals is to benefit the population by the wide adoption of various technologies. However, the effects remain statistically significant at some quantiles, although the impact varies across the different quantiles of WCI.

In addition, there are 3 continuous variables affecting the WCI. Specifically, the effect of the number of beds on WCI is positive for all quantiles but only significant from the 0.70 quantile to the 0.90 quantile, although the effect can no longer be found in the median level. Moreover, the total visiting number of outpatients and emergency patients and the activity index of WSVA both have a significant positive effect on WCI for all quantiles. Figure 7 also shows an overall upward trend, that is, the higher the quantile of WCI, the higher the positive effect of activity index on WCI, whereas the effect of the total visiting number of outpatients and emergencies shows fluctuation.

## Discussion

### Principal Findings

This study presented the distribution of WOAs operated by top tertiary hospitals in China and 4 clusters of the functional items of WSVA using CATPCA. The factors influencing the WCI were analyzed using quantile regression. The effects of the total number of visiting outpatients and emergency patients and the activity index of WSVA on the WCI are significant and positive for all quantiles. However, 3 dummy variables are seen to have no significant effect on the WCI.

First, the WOAs, especially WSVAs, have been widely adopted in top tertiary hospitals in China, indicating that they are supportive and aligned to the improvement of the efficiency for medical services and facilitating health care. To the best of the authors’ knowledge, one of the main reasons for this is that mobile reading and WeChat reading have already been included in the Chinese national reading category. According to the recent Chinese National Reading Survey, released by the China Press and Publication Research Institute, the proportion of WeChat reading has grown rapidly [55,56]. In addition, it seems that there are more provinces in the WC, but the number of WOAs is generally rare. However, after normalizing the number of WOAs operated by top tertiary hospitals targeted to every province in China, it can be found that there was no significant difference in WSSA adoption between the normalized value sets from WC, CC, and EC, with the *P* values more than .05 calculated using the Kruskal-Wallis test. All these results demonstrate that the wide application and mutual reference of mobile health technologies between tertiary hospitals make adoption of WOA similar.

In addition, there may still be some drawbacks in the application and promotion of WOAs. The QR code of the WOAs should be provided to facilitate patient scanning on the official website of the tertiary hospital. Some tertiary hospitals have not yet paid much attention to the application of WOAs, with uncreated or uncertified accounts or insufficient functions being provided. However, the WOAs will be an important part of communication if the hospital wants to improve its quality of medical services and influential power. In light of the trend reflected by the year of certification, the application of WOA to top tertiary hospitals is still in its infancy, which should be developed with more functional items and provide better services after certification.

Second, more functional items in the WSVAs should be developed to meet the various demands of patients. Currently, functional items of WSVAs are varied; the focus is generally on visiting appointments and inquiries, although there are 4 clusters of functional items observed. Another worthwhile function is medical charge payments. With the widespread popularity of mobile payment in China, online payments for medical services will undoubtedly be applied. However, with respect to security interface settings of WSVAs, there are still certain risks that have yet to be ameliorated.

Furthermore, it should be noted that interactive functions for medical services should be given more attention for development, although WOAs have increasingly integrated some functional items from mobile health care apps. With the improved expectation for medical service, some interactive functional items between doctors and patients should be strengthened or developed to further alleviate “the difficulties and high costs of obtaining medical services in China” and meet the higher medical service demands. The development of the functional item *online consultation* in the WSVA will further support patient-centered medical services. Certainly, there is a need for mechanisms to ensure that doctors participate in online counseling services in the context of the shortage of medical resources in China, for example, involving this service in the performance of doctors. The widespread application of such functions will alleviate the difficulty in seeing a doctor to some extent. This is consistent with *Healthy China 2030 Planning* [57], *Guidance on actively promoting the “Internet +” initiative* [58], *Opinions on Promoting the Development of “Internet + Medicine”* [59], and other relevant policies released by the State Council of China, which suggest speeding up the construction of smart hospitals to further facilitate patients.

Third, the top tertiary hospitals should post high-quality messages as much as possible to expand the influence of WOAs operated by top tertiary hospitals. As shown from the results of the above quantile regression analysis, the total number of visiting outpatients and emergency patients and activity index can promote the communication power of WSVAs. Therefore, it is necessary to take full advantage of the 4 times of posting, optimize the column settings of tweets, and improve the quality and originality of tweets that meet the demands of patients, which is similar to the operation of other social media in the health care field [60,61]. In addition, consumer health vocabulary and health literacy should also be developed and improved to help patients better understand messages [62-64], which will enhance the attractiveness and the degree of activity on WOAs. However, there are some top tertiary hospitals that have no WOAs. Accordingly, it is necessary for those hospitals without WOAs to set up a department or form a team to maintain the system and post high-quality messages. Therefore, all these measures will provide a valuable means to expand the influence of WSVAs and, in return, improve the patient experience, which is consistent with the aim of the reform of health care system in China.

In addition, CHs should also strengthen the construction of WSVAs, which typically apply to large tertiary hospitals at higher quantiles. First, the influence of the type of hospital on the WCI is statistically significant at the higher quantiles, suggesting that the type of tertiary hospital is a sensitive indicator of high WCI scores. From the above results, tertiary hospitals with different WCI scores should take the significant influence factors in the corresponding quantile regression into consideration to quickly improve the influence of their WSVAs. Second, the number of beds represents the size of the hospital. As the size of tertiary hospitals in China continues to expand, its impact on WCI decreases after the 0.70 quantile, the reason for which might be that the hospital staff might be busy and have no motivation to improve the impact of the WCI. Furthermore, tertiary hospitals with a large numbers of beds should take advantage of their size and develop chronic disease management and health education aimed at specific populations [65] by introducing new health care technologies using WSVAs, which will ease the conflicts between doctors and patients and establish a harmonious relationship between them.

### Limitations

Several potential limitations are worth noticing, and further research will be optimized on the basis of these. First, some bias might exist because we merely collected data from tertiary hospitals. Therefore, the results may not be truly representative of all hospitals in China. In addition, the hospital name and the name of WOA do not correspond in some instances, which means that some WOAs could not be found. Obviously, this affects the accuracy of the distribution of WOAs in China.

Second, the advantage of the CATPCA method is that it can grapple with various types of data, but these 14 functional items are summarized based on a certain amount of investigation and discussion, which might have effects on the categorization of the functional items of WSVA.

Finally, this study collected number of open beds as well as the total number of outpatients and emergency patients in 2017 from the homepages of tertiary hospitals. The time-lag updating of these data might lead to the bias of the result of quantile regression analysis. There also exists the different time of posting messages with the limitations of 4 times, but the relevant data were captured by Qingbo Index system at a fixed date, which may influence the result of the quantile regression analysis. Future research might be started based on panel data.

### Conclusions

In this study, the distribution of WOAs was presented. CATPCA was conducted with the data source from the WeChat app using SPSS 20.0. Quantile regression was used to explore and understand the factors that influenced the WCI, performed by the R 3.5.1. Obviously, quantile regression analysis can provide more abundant information by describing the different effects of independent variables on the dependent variable at different quantiles, which ensure better accuracy and robustness of the regression results. On the basis of the aforementioned results and discussion, some valuable and reasonable results for WOAs, including function constitution and influence factors on the WCI, were identified. With the widespread application of WOA, it is reasonable to believe that WOAs will be an important medium in the future to publicize hospitals and enhance the accessibility of medical care for patients, thereby improving patient experiences.

In addition, the results of this study are useful for tertiary hospitals for a better understanding of how to update the function of their WSVAs and improve the impact of their WCI. Specifically, this study’s results of the function constitution of WOAs will primarily help hospitals learn from each other. Subsequently, the hospitals, in partnership with the developer of WOAs, constantly optimize the interface and enrich the functional items of WOAs so as to form a complete, convenient, and integrated medical service for patients, which will alleviate the pressure of patients queuing for medical treatment. Meanwhile, on the basis of exploring the main factors influencing the communication of WOAs, the hospital may take targeted improvement measures to further enhance the timeliness, richness, and readability of health care–related information, which will further enlarge the hospital’s publicity on social media, cultivate positive usage intention, and increase the content experiences.

## Appendix

Multimedia Appendix 1 The detailed calculation formula of WeChat communication index.

Multimedia Appendix 2 The list of WeChat official accounts in all provinces of China.

Multimedia Appendix 3 Component loadings of 14 function items of WeChat service accounts.

Multimedia Appendix 4 The detailed results of quantile regression.

## Abbreviations

CATPCA: categorical principal components analysis
CC: Central China
CH: comprehensive hospital
EC: Eastern China
NHC-PRC: National Health Commission of the People’s Republic of China
OLS: ordinary least squares
QR: quick response
SH: specialized hospital
WC: Western China
WCI: WeChat communication index
WOA: WeChat official account
WSSA: WeChat subscription account
WSVA: WeChat service account
