# Supplementary material for: Understanding the Function Constitution and Influence Factors on Communication for the WeChat Official Account of Top Tertiary Hospitals in China: Cross-Sectional Study
Source: J Med Internet Res. 2019 Dec 9;21(12):e13025. doi: 10.2196/13025 (PMC6928700; doi:10.2196/13025)
Supplement: Multimedia Appendix 1 [file jmir_v21i12e13025_app1.doc]

**Multimedia Appendix 1: the detailed calculation formula of WeChat communication index.**

**1. The weight value of impact indicator of Wechat official account.**

| Primary index | Secondary index | Weight value |
| --- | --- | --- |
| Overall communication power  (30%) | Average daily reading (R/d) | 85% |
|  | Average daily click the “thumbs up” sign (Z/d) | 15% |
| Average dissemination power  (30%) | Average reading each article (R/n) | 85% |
|  | Average click the “thumbs up” sign each article (Z/n) | 15% |
| Headline communication power  (30%) | Average daily reading of headline (Rt/d) | 85% |
|  | Average daily click the “thumbs up” sign of headline (Zt/d) | 15% |
| Peak dissemination power  (10%) | Maximum reading (Rmax) | 85% |
|  | Maximum click the “thumbs up” sign (Zmax) | 15% |

**Note: R** is the total number of readings for all articles (n) in the evaluation period;

**Z** is the total number of click the “thumbs up” signs for all articles (n) in the evaluation period;

**d** is the days for assessment;

**n** is the number of articles posted in the assessment period;

**Rt** and **Zt** is the total number of readings and click the “thumbs up” signs for the headlines posted in the period of evaluation;

**Rmax** and **Zmax** is the highest number of reading and the best click the “thumbs up’’ sign for the articles posted in the period of evaluation.

**2. The Calculation formula of Wechat Communication Index.**

WCI={30%*[0.85*ln(R/d+1)+0.15*ln(10*Z/d+1)]+30%*[0.85*ln(R/n+1)+0.15*ln(10*Z/n+1)]

+30%[0.85*ln(Rt/d+1)+0.15*ln(10*Zt/d+1)]+10%*[0.85*ln(Rmax+1)+0.15*ln(10*Zmax+1)]}^2^*10
