# Supplementary material for: Understanding the Function Constitution and Influence Factors on Communication for the WeChat Official Account of Top Tertiary Hospitals in China: Cross-Sectional Study
Source: J Med Internet Res. 2019 Dec 9;21(12):e13025. doi: 10.2196/13025 (PMC6928700; doi:10.2196/13025)
Supplement: Multimedia Appendix 2 [file jmir_v21i12e13025_app2.doc]

**Multimedia Appendix 2: List of full name, abbreviation, number of WeChat subscription accounts and WeChat service accounts in all provinces of China.**

| Region | Full name of province | Abbreviation of province | Number of subscription accounts | Number of service accounts |
| --- | --- | --- | --- | --- |
| Western China | Nei Mongol Zizhiqu | NM | 5 | 7 |
|  | Shaanxi Sheng | SN | 12 | 13 |
|  | Ningxia Huizu Zizhiqu | NX | 0 | 3 |
|  | Gansu Sheng | GS | 1 | 10 |
|  | Qinghai Sheng | QH | 1 | 6 |
|  | Xinjiang Uygur Zizhiqu | XJ | 2 | 6 |
|  | Xizang Zizhiqu | XZ | 1 | 0 |
|  | Sichuan Sheng | SC | 4 | 29 |
|  | Chongqing Shi | CQ | 0 | 10 |
|  | Guizhou Sheng | GZ | 3 | 18 |
|  | Guangxi Zhuangzu Zizhiqu | GX | 7 | 17 |
|  | Yunnan Sheng | YN | 0 | 4 |
| Central China | Heilongjiang Sheng | HL | 12 | 15 |
|  | Jilin Sheng | JL | 7 | 11 |
|  | Shanxi Sheng | SX | 8 | 22 |
|  | Henan Sheng | HA | 7 | 15 |
|  | Anhui Sheng | AH | 1 | 18 |
|  | Hubei Sheng | HB | 1 | 32 |
|  | Hunan Sheng | HN | 2 | 18 |
|  | Jiangxi Sheng | JX | 3 | 30 |
| Eastern China | Liaoning Sheng | LN | 5 | 31 |
|  | Hebei Sheng | HE | 5 | 26 |
|  | Beijing Shi | BJ | 11 | 19 |
|  | Tianjin Shi | TJ | 8 | 9 |
|  | Shandong Sheng | SD | 3 | 18 |
|  | Jiangsu Sheng | JS | 6 | 32 |
|  | Shanghai Shi | SH | 0 | 24 |
|  | Zhejiang Sheng | ZJ | 4 | 21 |
|  | Fujian Sheng | FJ | 2 | 18 |
|  | Guangdong Sheng | GD | 3 | 57 |
|  | Hainan Sheng | HI | 1 | 4 |
