# Supplementary material for: Understanding the Function Constitution and Influence Factors on Communication for the WeChat Official Account of Top Tertiary Hospitals in China: Cross-Sectional Study
Source: J Med Internet Res. 2019 Dec 9;21(12):e13025. doi: 10.2196/13025 (PMC6928700; doi:10.2196/13025)
Supplement: Multimedia Appendix 3 [file jmir_v21i12e13025_app3.doc]

**Multimedia Appendix 3: component loadings of 14 function items of WeChat service accounts**

**Table** . Component loadings of 14 function items of WeChat service accounts.

| Function item | Dimension | | | |
| --- | --- | --- | --- | --- |
|  | 1 | 2 | 3 | 4 |
| Hospital_brief | -0.172 | 0.182 | 0.582 | 0.396 |
| Introduction_of_department_and_expert | -0.274 | -0.126 | 0.636 | -0.067 |
| Information_bulletin | -0.420 | -0.106 | 0.446 | -0.274 |
| Visiting_guide | 0.164 | 0.512 | 0.082 | -0.539 |
| Hospital_navigation | -0.001 | -0.100 | 0.498 | -0.043 |
| Visiting_appointment | 0.700 | -0.047 | 0.198 | -0.034 |
| Inquiry | 0.802 | -0.012 | 0.183 | 0.075 |
| Medical_charge_payment | 0.541 | -0.574 | 0.057 | -0.042 |
| Intelligent_guidance | 0.456 | 0.360 | -0.121 | 0.396 |
| Personal_information_management | 0.787 | -0.057 | 0.134 | -0.072 |
| Health_education | -0.050 | 0.681 | 0.124 | -0.083 |
| Advice_and_feedback | 0.312 | 0.547 | 0.084 | -0.171 |
| Related_links | 0.213 | -0.175 | 0.262 | -0.369 |
| Others | -0.022 | -0.132 | -0.302 | -0.579 |
