# Supplementary material for: Understanding the Function Constitution and Influence Factors on Communication for the WeChat Official Account of Top Tertiary Hospitals in China: Cross-Sectional Study
Source: J Med Internet Res. 2019 Dec 9;21(12):e13025. doi: 10.2196/13025 (PMC6928700; doi:10.2196/13025)
Supplement: Multimedia Appendix 4 [file jmir_v21i12e13025_app4.doc]

**Multimedia Appendix 4: the detailed results of quantile regression.**

The following 9 tables presents the coefficients estimates of quantile regression with WCI as dependent variable, with quantiles = 0.1, 0.2, 0.3, 0.4, 0.5, 0.6, 0.7, 0.8, 0.9.

**Quantile=0.10**

Coefficients:

Value Std. Error t value Pr(>|t|)

(Intercept) -359.04493 123.51514 -2.90689 0.00393

CertificationYear1 139.93338 142.69569 0.98064 0.32758

CertificationYear2 160.68162 109.54814 1.46677 0.14352

CertificationYear3 169.97525 112.10805 1.51617 0.13056

HospitalType1 -47.89635 38.85625 -1.23265 0.21870

ReformYear1 -17.66590 47.27945 -0.37365 0.70894

ReformYear2 52.61489 35.24239 1.49294 0.13654

ReformYear3 50.27342 38.97878 1.28976 0.19816

ReformYear4 16.41208 29.58689 0.55471 0.57952

BedNumber 12.72326 14.57811 0.87276 0.38351

TotalVisitingNumber 0.60192 0.14915 4.03558 0.00007

ActivityIndex 214.47357 19.46193 11.02016 0.00000

**Quantile=0.20**

Coefficients:

Value Std. Error t value Pr(>|t|)

(Intercept) -236.27731 95.13022 -2.48373 0.01356

CertificationYear1 154.29114 111.13532 1.38832 0.16610

CertificationYear2 56.23382 89.59029 0.62768 0.53071

CertificationYear3 63.40226 88.55397 0.71597 0.47458

HospitalType1 -71.90346 36.07228 -1.99332 0.04716

ReformYear1 60.07942 50.94901 1.17921 0.23928

ReformYear2 63.52656 33.56011 1.89292 0.05936

ReformYear3 73.53842 37.62797 1.95436 0.05162

ReformYear4 31.03822 36.23468 0.85659 0.39238

BedNumber 18.86153 14.96026 1.26078 0.20840

TotalVisitingNumber 0.74943 0.18662 4.01578 0.00008

ActivityIndex 215.32109 21.88486 9.83881 0.00000

**Quantile=0.30**

Coefficients:

Value Std. Error t value Pr(>|t|)

(Intercept) -261.07909 100.47025 -2.59857 0.00984

CertificationYear1 150.39655 113.77195 1.32191 0.18724

CertificationYear2 76.78358 95.79965 0.80150 0.42350

CertificationYear3 71.51867 97.29552 0.73507 0.46289

HospitalType1 -93.46224 40.08748 -2.33146 0.02041

ReformYear1 42.65419 45.42476 0.93901 0.34851

ReformYear2 57.92320 36.71470 1.57766 0.11573

ReformYear3 80.79198 39.02816 2.07009 0.03933

ReformYear4 41.59975 37.86050 1.09876 0.27278

BedNumber 8.72749 15.39920 0.56675 0.57132

TotalVisitingNumber 0.97993 0.18802 5.21192 0.00000

ActivityIndex 240.38441 21.83059 11.01136 0.00000

**Quantile=0.40**

Coefficients:

Value Std. Error t value Pr(>|t|)

(Intercept) -250.51859 90.35556 -2.77259 0.00592

CertificationYear1 129.62395 98.41539 1.31711 0.18884

CertificationYear2 84.85012 82.60026 1.02724 0.30516

CertificationYear3 78.80029 84.30591 0.93469 0.35072

HospitalType1 -105.99886 37.06503 -2.85981 0.00455

ReformYear1 47.16526 43.57063 1.08250 0.27993

ReformYear2 78.21504 37.84113 2.06693 0.03962

ReformYear3 69.89171 38.46125 1.81720 0.07022

ReformYear4 44.38049 36.77063 1.20695 0.22843

BedNumber 10.50107 15.56881 0.67449 0.50053

TotalVisitingNumber 1.04537 0.16098 6.49397 0.00000

ActivityIndex 245.77676 21.80022 11.27405 0.00000

**Quantile=0.50**

Coefficients:

Value Std. Error t value Pr(>|t|)

(Intercept) -237.15150 91.07444 -2.60393 0.00969

CertificationYear1 105.88323 106.46720 0.99451 0.32080

CertificationYear2 89.54662 80.94749 1.10623 0.26954

CertificationYear3 108.19484 84.21940 1.28468 0.19993

HospitalType1 -120.06275 37.88289 -3.16931 0.00169

ReformYear1 33.47225 40.15265 0.83362 0.40518

ReformYear2 41.22101 42.09767 0.97918 0.32831

ReformYear3 49.82040 36.18057 1.37699 0.16957

ReformYear4 32.52465 38.89769 0.83616 0.40375

BedNumber 14.90567 19.36690 0.76965 0.44213

TotalVisitingNumber 1.12076 0.17761 6.31023 0.00000

ActivityIndex 255.48604 20.63199 12.38301 0.00000

**Quantile=0.60**

Coefficients:

Value Std. Error t value Pr(>|t|)

(Intercept) -149.54841 93.51057 -1.59927 0.11085

CertificationYear1 0.68078 111.95129 0.00608 0.99515

CertificationYear2 0.98122 76.93986 0.01275 0.98983

CertificationYear3 15.65254 79.02583 0.19807 0.84313

HospitalType1 -107.63052 42.53541 -2.53037 0.01192

ReformYear1 26.35023 42.95124 0.61349 0.54003

ReformYear2 63.77742 41.44325 1.53891 0.12491

ReformYear3 44.37926 42.27062 1.04988 0.29464

ReformYear4 43.38979 35.77587 1.21282 0.22618

BedNumber 23.24008 22.42842 1.03619 0.30097

TotalVisitingNumber 1.05251 0.18389 5.72344 0.00000

ActivityIndex 265.47916 23.56016 11.26814 0.00000

**Quantile=0.70**

Coefficients:

Value Std. Error t value Pr(>|t|)

(Intercept) -194.79887 82.53973 -2.36006 0.01893

CertificationYear1 68.10560 100.91200 0.67490 0.50027

CertificationYear2 53.65848 59.58431 0.90055 0.36857

CertificationYear3 71.40736 62.22549 1.14756 0.25209

HospitalType1 -160.47973 39.42688 -4.07031 0.00006

ReformYear1 5.87427 57.59580 0.10199 0.91883

ReformYear2 61.78381 39.56813 1.56145 0.11950

ReformYear3 52.62121 50.79402 1.03597 0.30108

ReformYear4 57.60655 41.63758 1.38352 0.16756

BedNumber 41.54945 22.77963 1.82397 0.06918

TotalVisitingNumber 0.95687 0.18350 5.21449 0.00000

ActivityIndex 296.97460 23.20945 12.79542 0.00000

**Quantile=0.80**

Coefficients:

Value Std. Error t value Pr(>|t|)

(Intercept) -224.43963 83.31551 -2.69385 0.00747

CertificationYear1 53.41994 88.37204 0.60449 0.54599

CertificationYear2 37.08927 54.52837 0.68018 0.49693

CertificationYear3 74.09456 57.56740 1.28709 0.19908

HospitalType1 -133.03562 37.08173 -3.58763 0.00039

ReformYear1 26.39290 69.91794 0.37748 0.70609

ReformYear2 69.26097 46.64416 1.48488 0.13866

ReformYear3 46.21469 51.12792 0.90390 0.36679

ReformYear4 64.76732 46.68292 1.38739 0.16639

BedNumber 37.90595 18.31409 2.06977 0.03936

TotalVisitingNumber 1.08751 0.17732 6.13292 0.00000

ActivityIndex 324.47537 26.27496 12.34922 0.00000

**Quantile=0.90**

Coefficients:

Value Std. Error t value Pr(>|t|)

(Intercept) -146.08407 88.57417 -1.64929 0.10017

CertificationYear1 184.93091 111.56888 1.65755 0.09849

CertificationYear2 125.36522 57.73158 2.17152 0.03070

CertificationYear3 145.21932 56.81438 2.55603 0.01110

HospitalType1 -147.51444 36.44048 -4.04809 0.00007

ReformYear1 23.34340 80.33772 0.29057 0.77159

ReformYear2 30.07572 56.08658 0.53624 0.59220

ReformYear3 30.51465 56.23168 0.54266 0.58778

ReformYear4 6.17221 51.55324 0.11973 0.90478

BedNumber 17.97767 17.14461 1.04859 0.29524

TotalVisitingNumber 1.16072 0.17251 6.72829 0.00000

ActivityIndex 307.85611 29.19847 10.54357 0.00000
